# Urinary sC5b-9 is Better Linked to Albuminuria Than to Intrarenal Inflammation in Common Kidney Disease

**DOI:** 10.1016/j.ekir.2024.08.009

**Published:** 2024-08-13

**Authors:** Sébastien Kissling, Nora Schwotzer, Mireille Moser, Marc Froissart, Fadi Fakhouri

**Affiliations:** 1 Service of Nephrology and Hypertension, CHUV, Lausanne University, Lausanne, Switzerland; 2 Clinical Research Center, University Hospital of Lausanne and University of Lausanne, Switzerland; 3 Research and Education Department, University Hospital of Lausanne and University of Lausanne, Switzerland

**Keywords:** albuminuria, complement, inflammation, sC5b-9

## Introduction

The availability of an expanding number of complement inhibitors has renewed the interest in the role of complement in various kidney diseases. This has also highlighted the need for biomarkers that help clinicians assess renal complement activation and ensuing inflammation and monitor treatment effects. Several complement biomarkers in blood, urine, and kidney biopsy (KB) are available.^1^^,^^2^^,^S1 Bb/Ba and sC5b-9, markers of the activation of the complement alternative and final pathways respectively, are frequently used as complement biomarkers in various clinical settings and studies. In kidney diseases, analysis of urine provides a unique opportunity to explore intrarenal complement activation. We prospectively assessed the clinical value of urinary sC5b-9 (usC5b-9) and Bb (uBb) as biomarkers of intra-renal inflammation and complement deposition.

## Results

Patients’ main clinical and biological characteristics are summarized in Table 1 (full details are provided in Supplementary Table S1**)**. All patients had undergone KB for a clinical indication. Of the patients, 56.5% had glomerular diseases (including 11.4% with diabetic kidney disease), and 23.4% and 20.2% had tubulointerstitial and vascular nephropathies, respectively. Median estimated glomerular filtration rate (Chronic Kidney Disease: Epidemiology 2021 formula) was 43 ml/min per 1.73 m^2^. Median urinary albumin-to-creatinine ratio was 106 mg/mmol.

**Table 1:** Main characteristics of 124 patients included in the study

Features	*N* = 124
Female	58 (46.8%)
Age (yr)	59.0 (41.5–72.0)
Obesity (BMI ≥ 30 kg/m^2^)	24 (19.4%)
Diabetes mellitus	18 (14.5%)
Serum creatinine (μmol/l)	145 (96–209)
eGFR according to CKD-EPI (ml/min per 1.73 m^2^)	43.0 (28.0–66.5)
Urinary albumin-to-creatinine ratio (mg/mmol)	106 (15.3–314)
Nephrotic-range proteinuria (> 3 g/d)	36 (29%)
Type of nephropathy	
Glomerular	70 (56.5%)
Tubulointerstitial	29 (23.4%)
Vascular	25 (20.2%)
Presence of glomerular immune deposits	40 (32.3%)^a^
Crescentic glomerulonephritis	18 (14.5%)
Glomerulosclerosis score	
0	40 (26.4%)
1	42 (27.8 %)
2	17 (11.3%)
3	25 (16.6%)
Interstitial fibrosis score	
0	29 (17.5%)
1	38 (22.9%)
2	43 (25.9%)
3	14 (8.4%)
Serum sC5b-9 (ng/ml)	181 (142–266)
Serum Bb (mg/ml)	1.15 (0.88–1.60)
Detectable urinary sC5b-9^a^	56 (45.2%)
Detectable urinary Bb^a^	40 (32.3%)
Within subgroups with detectable values:	
Urinary sC5b-9 (ng/ml)	31.5 (15.3–125.8)
Urinary sC5b-9/cr (ng/mmol)	6.53 (2.59–15.3)
Urinary Bb (mg/ml)	0.365 (0.115–0.848)
Urinary Bb/Cr (mg/mmol)	0.057 (0.024–0.234)

CKD-EPI, Chronic Kidney Disease: Epidemiology formula; eGFR, estimated glomerular filtration rate.

Presented results are count (percentage) for categorical variables and median (1^st^ quartile–3^rd^ quartile) for continuous variables.

a The limit of detection of sC5b-9 and Bb is 3.7 ng/ml and 0.018 μg/ml, respectively; and the lower limit of quantification of sC5b-9 and Bb is 8.8 ng/ml and 0.033 μg/ml, respectively (according to the manufacturer’s notice).

uBb and usC5b-9 were detectable in the urine of 32.5% and 45.2% of patients, respectively; with median uBb/uCr and usC5b-9/uCr ratio of 0.057 mg/mmol and 6.53 ng/mmol, respectively. uBb/uCr ratio and usC5b9/uCr ratio did not correlate (*P*-value > 0.01, Kendall tau < 0.25 for all ordered variables) with age, sex, presence of obesity and diabetes, type of nephropathy, serum creatinine, renal inflammatory and fibrosis scores in KB, or the presence of immune renal (glomerular) deposits. The variable which displayed the strongest correlation with both usC5b-9/uCr ratio, and uBb/uCr ratio, is urinary albumin-to-creatinine ratio (Supplementary Table S2). Albuminuria and not proteinuria was used in the analysis because albuminuria is more widely accepted as a surrogate marker for kidney disease progression. Besides, proteinuria includes all complement components; thus, it is not fully independent from complement urinary biomarkers.

## Discussion

Our data indicate that usC5b-9 and uBb are not consistent biomarkers of intrarenal (complement-mediated) inflammation and of complement fragments deposition (C3, C4, and C1q). The source of usC5b-9 remains unclear. Unlike uBb, sC5b-9 has a high molecular weight (∼ 1000 KDa) and is thus not filtered by glomeruli even in the setting of heavy proteinuria. In previous studies in patients with nephrotic syndrome, serum and urinary levels of sC5b-9 did not correlate,^3^ and kidney function impairment does not affect sC5b-9 serum levels.^4^ Our results suggest that proteinuria is a major driver of intrarenal complement activation, even in nephropathies not characterized by complement glomerular deposition. This hypothesis is supported by experimental findings indicating that proteinuria enhances complement alternative pathway activation through the promotion of the docking of properdin (a stabilizer of the alternative C3 convertase) to the apical surface of proximal tubular cells^5^ and the impairment of factor H binding to these cells.^6^ sC5b-9 staining has not been performed in the KBs of the included patients. However, this marker is not fully reliable because staining for sC5b-9 in kidney tissue may be negative in a substantial proportion of patients with atypical hemolytic uremic syndrome, a prototypic disease mediated by C5 activation.^7^ Furthermore, sC5b-9 staining may persist in kidney tissue several months after the resolution of complement activation.^8^

Therefore, the assessment of complement involvement and of the impact of complement inhibitors in kidney diseases, should be dual. The first aspect relates to the specific role of complement as a primary driver of (mainly glomerular) kidney diseases. The second concerns the nonspecific proteinuria-induced complement activation. Both aspects may contribute to the progression of kidney diseases because complement activation induces podocyte and mitochondrial dysfunction^9^^,^S1 and activates interstitial profibrotic pathways.S2,S3 The C5 blocker, eculizumab, is detected in the urine at significant concentrations in the setting of heavy proteinuria;S4 however, the potential clinical benefit of complement inhibition to prevent proteinuria-induced kidney damage, remains to be established. Nevertheless, our data indicate that the urinary biomarkers, usC5b-9 and uBb, may reflect mainly proteinuria-induced nonspecific complement activation.

Our study has some limitations. It included a heterogenous group of nephropathies, with a relatively small subset of patients with renal inflammatory changes and used only 1 type of commercially available kits for the measurement of sC5b-9 and Bb. Furthermore, it did not include rare prototypic complement-mediated kidney diseases, such as atypical hemolytic uremic syndrome or C3 glomerulopathy, and the usefulness of usC5b-9 and uBb in these peculiar nephropathies remains to be assessed. However, more than half of the patients had glomerular diseases, approximately one-third had nephrotic-range proteinuria, and the study was designed to assess the clinical value of these 2 complement biomarkers in routine settings. Finally, markers of activation of the classical and lectin pathways have not been assessed. Nevertheless, our data, generated in a real-life setting, plead for caution in the analysis of complement biomarkers in common kidney diseases, most particularly in the era of complement inhibitors.

## Disclosure

All the authors declared no competing interests.

## Supplementary Material

Supplementary File (PDF) **Kidney pathology score. Statistical Methods. Supplementary References. Table S1.** Characteristics of included patients. **Table S2.** Statistical analysis of the correlation between uSc5b-9 and uBb and clinical, **biological, and pathological parameters.**

## Footnotes

Supplementary File (PDF)

**Kidney pathology score.**

**Methods.**

**Supplementary References.**
**Table S1**. Characteristics of included patients. **Table S2**. Statistical analysis of the correlation between uSc5b-9 and uBb and clinical, biological, and pathological parameters.
