# Supplementary material for: Urinary sC5b-9 is Better Linked to Albuminuria Than to Intrarenal Inflammation in Common Kidney Disease
Source: Kidney Int Rep. 2024 Aug 13;9(11):3335–7. doi: 10.1016/j.ekir.2024.08.009 (PMC11551109; doi:10.1016/j.ekir.2024.08.009)
Supplement: Supplementary File (PDF) — Kidney pathology score. Statistical Methods. Supplementary References. Table S1. Characteristics of included patients. Table S2. Statistical analysis of the correlation between uSc5b-9 and uBb and clinical, biological, and pathological parameters. [file mmc1.pdf]

| | usC5b-9 ≤ LOD (n=68) | usC5b-9 > LOD (n=56) | Overall (n=124) |
|---|---|---|---|
| *Patients characteristics* | | | |
| **Female** | 36 (52.9%) | 22 (39.3%) | 58 (46.8%) |
| **Age (years)** | 55.5 [39.0; 71.0] | 59.5 [45.5; 72.5] | 59.0 [41.5; 72.0] |
| **Obesity (BMI ≥ 30 kg/m²)** | 16 (23.5%) | 8 (14.3%) | 24 (19.4%) |
| **Diabetes Mellitus** | 9 (13.2%) | 9 (16.1%) | 18 (14.5%) |
| **Serum creatinine (□mol/L)** | 146 [93; 195] | 145 [101; 227] | 145 [96; 209] |
| **Estimated glomerular filtration rate** | 42.5 [30.0; 71.8] | 43.5 [25.0; 59.0] | 43.0 [28.0; 66.5] |
| **Urinary albumin to creatinine ratio** | 30.0 [5.8; 108.0] | 205.0 [131.0; 649.0] | 106.0 [15.3 ; 314.0] |
| **Serum sC5b-9** | 168 [124; 242] | 210 [164; 316] | 181 [142; 266] |
| **Serum Bb** | 1.12 [0.87; 1.52] | 1.18 [0.91; 1.77] | 1.15 [0.88; 1.60] |
| | | | |
| *Renal pathology features* | | | |
| **Type of nephropathy** | | | |
| **Glomerular** | 32 (47.1%) | 38 (67.9%) | 70 (56.5%) |
| **Tubulo-interstitial** | 23 (33.8%) | 6 (10.7%) | 29 (23.4%) |
| **Vascular** | 13 (19.1%) | 12 (21.4%) | 25 (20.2%) |
| | | | |
| **Glomerular immune deposits** | 19 (27.9%) | 21 (37.5%) | 40 (32.3%)* |
| | | | |
| *Activity_score (0-12)** | | | |
| **0** | 29 (42.6%) | 30 (53.6%) | 59 (47.6%) |
| **1** | 18 (26.5%) | 7 (12.5%) | 25 (20.2%) |
| **2** | 10 (14.7%) | 6 (10.7%) | 16 (12.9%) |
| **3** | 8 (11.8%) | 4 (7.1%) | 12 (9.7%) |
| **4** | 0 (0%) | 4 (7.1%) | 4 (3.2%) |
| **5** | 2 (2.9%) | 2 (3.6%) | 4 (3.2%) |
| **6** | 1 (1.5%) | 1 (1.8%) | 2 (1.6%) |
| **7** | 0 (0%) | 1 (1.8%) | 1 (0.8%) |
| **8** | 0 (0%) | 1 (1.8%) | 1 (0.8%) |
| *Activity score components* | | | |
| **Crescent score** | | | |
| **0** | 61 (89.7%) | 45 (80.4%) | 106 (85.5%) |
| **1** | 2 (2.9%) | 3 (5.4%) | 5 (4.0%) |
| **2** | 3 (4.4%) | 3 (5.4%) | 6 (4.8%) |
| **3** | 2 (2.9%) | 5 (8.9%) | 7 (5.6%) |
| **Endocapillary hypercellularity score** | | | |
| **0** | 63 (92.6%) | 46 (82.1%) | 109 (87.9%) |
| **1** | 5 (7.4%) | 6 (10.7%) | 11 (8.9%) |
| **2** | 0 (0%) | 1 (1.8%) | 1 (0.8%) |
| **3** | 0 (0%) | 3 (5.4%) | 3 (2.4%) |
| **Mesangial hypercellularity score** | | | |
| **0** | 56 (82.4%) | 48 (85.7%) | 104 (83.9%) |
| **1** | 10 (14.7%) | 3 (5.4%) | 13 (10.5%) |
| **2** | 2 (2.9%) | 4 (7.1%) | 6 (4.8%) |
| **3** | 0 (0%) | 1 (1.8%) | 1 (0.8%) |

| | | | |
|---|---|---|---|
| **Interstitial_inflammatory score** | | | |
| **0** | 40 (58.8%) | 38 (67.9%) | 78 (62.9%) |
| **1** | 16 (23.5%) | 14 (25.0%) | 30 (24.2%) |
| **2** | 7 (10.3%) | 3 (5.4%) | 10 (8.1%) |
| **3** | 5 (7.4%) | 1 (1.8%) | 6 (4.8%) |
| | | | |
| ***Chronicity score (0-7)*** | | | |
| **0** | 8 (11.8%) | 8 (14.3%) | 16 (12.9%) |
| **1** | 11 (16.2%) | 14 (25.0%) | 25 (20.2%) |
| **2** | 13 (19.1%) | 6 (10.7%) | 19 (15.3%) |
| **3** | 11 (16.2%) | 4 (7.1%) | 15 (12.1%) |
| **4** | 16 (23.5%) | 5 (8.9%) | 21 (16.9%) |
| **5** | 6 (8.8%) | 9 (16.1%) | 15 (12.1%) |
| **6** | 3 (4.4%) | 6 (10.7%) | 9 (7.3%) |
| **7** | 0 (0%) | 4 (7.1%) | 4 (3.2%) |
| ***Chronicity score components*** | | | |
| **Glomerulosclerosis score** | | | |
| **0** | 22 (32.4%) | 18 (32.1%) | 40 (32.3%) |
| **1** | 23 (33.8%) | 19 (33.9%) | 42 (33.9%) |
| **2** | 12 (17.6%) | 5 (8.9%) | 17 (13.7%) |
| **3** | 11 (16.2%) | 14 (25.0%) | 25 (20.2%) |
| **Interstitial fibrosis score** | | | |
| **0** | 13 (19.1%) | 16 (28.6%) | 29 (23.4%) |
| **1** | 25 (36.8%) | 13 (23.2%) | 38 (30.6%) |
| **2** | 23 (33.8%) | 20 (35.7%) | 43 (34.7%) |
| **3** | 7 (10.3%) | 7 (12.5%) | 14 (11.3%) |
| **Vascular score** | | | |
| **0** | 58 (85.3%) | 34 (60.7%) | 92 (74.2%) |
| **1** | 10 (14.7%) | 22 (39.3%) | 32 (25.8%) |

**Supplemental Table S1**: Characteristics of included patients with detectable or undetectable urinary sC5b-9.

Abbreviations: LOD, limit of detection (3.7 ng/mL for sC5b-9).

* Activity score empty classes 9 to 12 are not reported

| | usC5b-9 / uCr | | uBb -9 / uCr | |
|---|---|---|---|---|
| | τ or median by group | p-value | τ or median by group | p-value |
| **Sex** | 0.66 - 0 | 0.165 | 0 - 0 | 0.150 |
| **Age** | 0.07 | 0.291 | 0.14 | 0.038 |
| **Obesity** | 0 - 0 | 0.168 | 0 - 0 | 0.925 |
| **Diabetes mellitus** | 0 - 0.28 | 0.517 | 0 - 0 | 0.190 |
| **Serum creatinine** | 0.04 | 0.548 | 0.10 | 0.166 |
| **eGFR** | -0.05 | 0.478 | -0.11 | 0.103 |
| **Urinary ACR** | 0.48 | <0.001 | 0.44 | <0.001 |
| **Serum sC5b-9** | 0.17 | 0.011 | 0.15 | 0.036 |
| **Serum Bb** | 0.03 | 0.712 | 0.13 | 0.073 |
| **Type of nephropathy** | 1.06 - 0 - 0 | 0.015 | 0 - 0 - 0 | 0.098 |
| **IgG deposits** | 0.13 | 0.097 | 0.03 | 0.749 |
| **IgA deposits** | -0.07 | 0.370 | -0.08 | 0.357 |
| **IgM deposits** | 0.06 | 0.464 | -0.05 | 0.548 |
| **c3 deposits** | 0.03 | 0.714 | 0.05 | 0.572 |
| **c1q deposits** | -0.09 | 0.252 | -0.04 | 0.628 |
| **Any glomerular immune deposit** | 0.05 | 0.503 | 0.04 | 0.613 |
| **Activity score** | -0.08 | 0.259 | -0.11 | 0.169 |
| **Crescents score** | 0.01 | 0.892 | -0.14 | 0.086 |
| **Interstitial inflammation score** | -0.12 | 0.120 | -0.07 | 0.416 |
| **Endocapillary hypercellularity score** | 0.12 | 0.145 | 0.07 | 0.433 |
| **Mesangial hypercellularity score** | -0.06 | 0.468 | -0.09 | 0.288 |
| **Chronicity score** | 0.06 | 0.427 | 0.15 | 0.047 |
| **Glomerulosclerosis score** | 0.04 | 0.598 | 0.08 | 0.325 |
| **Interstitial fibrosis score** | 0.02 | 0.831 | 0.18 | 0.018 |
| **Vascular score** | 0.25 | 0.002 | 0.24 | 0.004 |

**Supplemental Table S2**: Kendall correlation (τ) and associated p-value for ordered variables. For categorical non-ordered variables, median by group are presented as well as the p-value of exact Wilcoxon test or Kruskall-Wallis. No correction for multiplicity of test is applied.

**Kidney pathology scores**

Histopathology score was adapted from the C3 glomerulopathy histopathology index devised by Bomback et al[S6]. The index includes both activity and chronicity scoring categories.

**Activity score (0-12)**.

Crescent score, Crescent formation score corresponding to % of glomeruli with cellular and/or fibrocellular crescents (0% = 0; 1-10% = 1; 11-25% = 2; > 25% = 3).

Endocapillary hypercellularity score, Endocapillary hypercellularity/proliferation corresponding to % of glomeruli with increased number of cells within glomerular capillary lumina causing luminal narrowing (none = 0; 1-25% = 1; 26-50% = 2; > 50% = 3).

Mesangial hypercellularity score, Mesangial hypercellularity corresponding to % of glomeruli with > 3 mesangial cells per mesangial area (none = 0; 1-25% = 1; 26-50% = 2; > 50% = 3).

Interstitial inflammatory score, Interstitial inflammation score corresponding to % of cortical tubulointerstitial area of inflammation in non-fibrotic cortex (< 10% = 0; 10-25% = 1; 26-50% = 2; > 50% = 3).

**Chronicity score (0-7)**.

Glomerulosclerosis score, Glomerulosclerosis corresponding to % of glomeruli with global sclerosis (< 10% = 0; 10-25% = 1; 26-50% = 2; > 50% = 3).

Interstitial fibrosis score, corresponding to % of cortical tubulointerstitial area involved with interstitial fibrosis (< 10% = 0; 10-25% = 1; 26-50% = 2; > 50% = 3).

Vascular score, grading the degree of arterial and arteriolar lesions (0 or 1).

**Methods**

Between the 1st of January 2021 and the 31st of January 2023, 124 consecutive patients undergoing a kidney biopsy (KB) in the department of Nephrology and Hypertension at CHUV, Lausanne, were prospectively included in the study. For all patients, urine samples were collected at the time of KB (two hours before the procedure). Samples were stored on ice, immediately transfered to the immunology laboratory with a measurement done in routine within 2 hours, without any storage, freezing and thawing processes that may induce complement activation ex vivo. uBb and usC5b-9 levels were measured using commercially available kits (Quidel, San Diego, CA, USA) and expressed as a ratio to urine creatinine (uCr): uBb/uCr ratio and usC5b-9/uCr ratio in □g/mmol and ng/mmol, respectively. All patients gave informed consent for inclusion in the study that was approved by the local ethics committee

(CER-VD; number 2023-00410). Relevant clinical data were extracted from patients' medical records. KB were blindly reviewed, and inflammatory and fibrotic changes were scored using a semi-quantitative scale (see Kidney pathology score in Supplemental Data).

Categorical data were described by count and frequencies, continuous data were described by 1st quartile, median and 3rd quartile. uBb/uCr and usC5b9/uCr ratio cannot be computed for values below LOD. Values below LOD were imputed to 0 (the choice of this value is not important as statistical analysis relies only on non-parametric approach). To assess the correlation uBb/uCr and usC5b9/uCr ratio and other variables, Kendall correlation (τ) and associated p-value for ordered variable are reported. For categorical non-ordered values, the median of uBb/uCr and usC5b9/uCr by group are presented as well as the p-value of exact Wilcoxon test or Kruskall-Wallis. No correction for multiplicity of tests was applied, however, in order to avoid too many false positive a threshold of 0.01 was chosen.

Additionally, data were split in two groups: the one with a detectable level of usC5b-9 and the one without. Characteristics of these groups were described by count and frequencies, continuous data were described by 1st quartile, median and 3rd quartile.

.Analysis was performed with R version 4.3.0 (2023-04-21 ucrt) [S7]. R packages Kendall[S8], pRoC[S9] and coin[S10] were used.

## Supplemental References


S1. Wijaya C, Burns C, Hall S, et al. Measurement of complement activation via plasma soluble C5b-9 comparison with terminal complement complex staining in a series of kidney biopsies. Kidney Blood Press Res 2023;48(1):220-30. DOI: 10.1159/000529734.

S2. Tan SM, Ziemann M, Thallas-Bonke V, et al. Complement C5a Induces Renal Injury in Diabetic Kidney Disease by Disrupting Mitochondrial Metabolic Agility. Diabetes 2020;69(1):83-98. DOI: 10.2337/db19-0043.

S3. Martin IV, Bohner A, Boor P, et al. Complement C5a receptors C5L2 and C5aR in renal fibrosis. Am J Physiol Renal Physiol 2018;314(1):F35-F46. DOI: 10.1152/ajprenal.00060.2017.

S4. Abbate M, Zoja C, Corna D, et al. Complement-mediated dysfunction of glomerular filtration barrier accelerates progressive renal injury. J Am Soc Nephrol 2008;19(6):1158-67. DOI: 10.1681/ASN.2007060686.

S5. Wijnsma KL, Ter Heine R, Moes D, et al. Pharmacology, Pharmacokinetics and Pharmacodynamics of Eculizumab, and Possibilities for an Individualized Approach to Eculizumab. Clin Pharmacokinet 2019;58(7):859-874. DOI: 10.1007/s40262-019-00742-8.

S6. Bomback AS, Santoriello D, Avasare RS, et al. C3 glomerulonephritis and dense deposit disease share a similar disease course in a large United States cohort of patients with C3 glomerulopathy. Kidney Int 2018;93(4):977-985. DOI: 10.1016/j.kint.2017.10.022.
S7. Team RC. R: A Language and Environment for Statistical Computing_. R Foundation for Statistical Computing, Vienna, Austria. 2023:https://www.R-project.org/.
S8. (2022) MA. Kendall: Kendall Rank Correlation and Mann-Kendall Trend Test_. R package version 2.2.1. 2022(https://CRAN.R-project.org/package=Kendall).
S9. Robin X, Turck N, Hainard A, et al. pROC: an open-source package for R and S+ to analyze and compare ROC curves. BMC Bioinformatics 2011;12:77. DOI: 10.1186/1471-2105-12-77.
S10. Hothorn T HK, van de Wiel MA, Zeileis A A Lego system for conditional inference. The American Statistician 2006;60(3):257-263.
